# Construction of women's empowerment index for Bangladesh

**DOI:** 10.3389/fsoc.2024.1356756

**Published:** 2024-11-12

**Authors:** Shanjida Chowdhury, Md. Mehedi Hasan Khan, Md. Aminul Haque

**Affiliations:** ^1^Department of Population Sciences, University of Dhaka, Dhaka, Bangladesh; ^2^Southeast University, Dhaka, Bangladesh

**Keywords:** women's empowerment, gender equality, exploratory factor analysis, confirmatory factor analysis, Bangladesh

## Abstract

**Introduction:**

This study is dedicated to refining and enhancing the measurement model of women's empowerment in Bangladesh. Women's empowerment, a crucial and multifaceted aspect of societal growth, is often hindered by gender disparities. This is particularly evident in societies like Bangladesh, where women face inequalities in education, economic opportunities, and decision-making power. To address these disparities effectively, it is essential to have a comprehensive understanding of women's empowerment. Therefore, this study aims to refine and enhance the measurement model to capture the multifaceted nature of women's empowerment accurately.

**Methods:**

To gather data for this study, a structured questionnaire was administered to married women of reproductive age (15–49) in eight Mouza/Mohalla in Dhaka, Bangladesh. This unique approach allowed us to capture a diverse range of perspectives. We used thirty-three indicators across economic, socio-cultural, household, and psychological dimensions to measure women's empowerment. The sample data were then randomly divided for exploratory factor analysis (EFA) and Confirmatory Factor Analysis (CFA) to identify and validate a comprehensive multidimensional framework.

**Results:**

Out of 625 respondents, only 36% of women worked, and ~39% married before age 18. Employing thirty-three items in EFA led to identifying eight critical factors (economic independence, control over household financial decisions, household decision-making, reproductive decision-making, freedom of movement, media exposure, positive self-esteem, and negative self-esteem). These factors, which explained 72.661% of the total variance in the data, provide a practical framework for understanding and addressing women's empowerment. Each component was then divided into two sub-dimensions to acquire a better understanding. The CFA indicated a good model fit for each dimension, and convergent and discriminant validity assessments were used to establish reliability and validity, further enhancing the practical implications of our findings.

**Conclusions:**

The results of our rigorous exploratory and confirmatory factor analyses not only confirmed the sample structures and internal consistency but also provided significant insights. The findings suggested an adequate fit for all CFA models, indicating the robustness of our measurement model. According to the CFA results, each dimension's variables are satisfactory, and all the dimensions can be combined to create a single index measuring women's empowerment. This comprehensive understanding of women's empowerment, with its specific dimensions and factors, equips policymakers and practitioners with the knowledge to develop a wide range of interventions appropriate to particular facets of empowerment, thereby fostering societal growth and gender equality.

## Introduction

Women's empowerment is not just a crucial pathway for achieving gender equality. It is the foundation upon which these goals are built, including the Sustainable Development Goals (SDGs). Promoting women's rights and reversing unequal power relations between males and females are not just essential stages in the process of empowerment; they are the key to unlocking a future of better health, better education, more chances for employment, and higher participation in decision-making both inside and outside of homes. It is a social and self-transformation process that provides women with power, control, and meaningful choices in their lives, and it is this empowerment that will drive us toward a more equal and sustainable future.

The concept of women's empowerment has evolved, reflecting women's diverse experiences and perspectives across different contexts. It is a complex process that requires a holistic approach, addressing individual and societal levels. In previous literature, women's empowerment has been conceptualized in terms of power (Mason, 1995; Mason and Smith, 2003), freedom (Narayan-Parker, 2002; Sen and Batliwala, 2000), autonomy (Malhotra and Schuler, 2005), decision-making, mobility (Narayan-Parker, 2002; Sen and Batliwala, 2000; Rodwell, 1996), self-efficacy (Upadhyay et al., 2014; Gram et al., 2019), income (Kabir et al., 2019), and self-compassion (Samanta, 2020). Empowerment has been defined differently across various academic literature, often in conjunction with the concepts of power, resources, agency, opportunity, and choice (Rowlands, 1997; Malhotra et al., 2002; Alsop and Heinsohn, 2005; Ibrahim and Alkire, 2007).

Measuring women's empowerment (MoE) is also challenging because it incorporates multidimensional aspects of life, including the age at which significant life events like marriage or the birth of a child occur, educational attainment, occupational status (Radovic Markovic and Achakpa, 2018), how partners view and discuss family planning, freedom of movement, and political representation (Ewerling et al., 2017; Phan, 2016). Researchers have encountered significant difficulties in quantifying and comparing the variables across various contexts to measure women's empowerment (Huis et al., 2017; Miedema et al., 2018; Richardson, 2018; Laszlo et al., 2020; Peterman et al., 2021). Initiatives have been taken to simplify the MoE by capturing key characteristics: control over resources, decision-making ability, societal status, and knowledge (Richardson, 2018; Laszlo et al., 2020; Bayeh, 2016; Ewerling et al., 2018; Soharwardi and Ahmad, 2020). The research question for this study is to know how WE can be measured using social, household, socio-cultural, and psychological dimensions.

### Theoretical framework

The theoretical framework of women's empowerment includes diverse sociological, psychological, and economic perspectives that address how women gain power and autonomy. The framework focuses on essential principles such as resources, agency, awareness of gender-based inequalities, local and global socio-economic structures, and the intersectionality of gender with other social categories (e.g., race, class, ethnicity) (Kabeer, 1999). The capability approach significantly influences this framework by focusing on the freedom to achieve desired outcomes, underlining the importance of providing women with opportunities and resources (Sen, 1987, 1993). Other frameworks emphasize the economic, socio-cultural, familial/interpersonal, psychological, legal, technological (Kabir et al., 2023), and political dimensions of women's lives (Malhotra et al., 2002).

Indexes also measure women's inequality, empowerment, and parity. These are the Gender Inequality Index (GII) measures gender disparities in reproductive health, empowerment, and labor force participation (UNDP, 2010), and the Gender Development Index (GDI) evaluates gender disparities in human development (health, knowledge, and living standards), are two indices that are used internationally (UNDP, 1995a). The Women's Empowerment Index (WEI) deals with Life and good health, education, skill-building knowledge, labor and financial inclusion (United Nations Development Programme, 2023), participation in decision-making, and freedom from violence. The Global Gender Gap Index (GGI) assesses women's empowerment in five primary domains: educational achievement, health and wellbeing, economic opportunities, economic participation, and political empowerment (United Nations Development Programme, 2023). The Global Gender Index (GGI) uses four indicators: economic participation and opportunities, educational attainment, health and survival, and political participation (Forum WE, 2023). The GEM assesses women's empowerment on a broader scale by utilizing a limited number of indicators and emphasizing income (UNDP, 1995b). Broadly, all the indexes deal with the macro level 4–10 indicators on economic, educational, health, employment, political participation, living standards, etc. The indexes used secondary-level data and prepared a country-level gender index.

To comprehend the multidimensionality of empowerment, researchers used combinations of multiple factors beyond the macro-level variables to describe empowerment in different sociocultural contexts (Peterman et al., 2021; Bastagli et al., 2016). The research incorporated the six categories, like mobility, economic security, ability to influence family decisions, effectiveness in the public sector, and non-family cluster, contributing to measuring women's empowerment (Schuler and Hashemi, 1994). Another study revealed four dimensions of empowerment: self-esteem, involvement in household decision-making, freedom of movement, and resource control (Mahmud et al., 2012). Studies measured women's empowerment using economic, sociocultural, legal, political, and psychological dimensions (Malhotra and Schuler, 2005; Malhotra et al., 2002; Khan et al., 2020). Decision-making, knowledge, physical, emotional, economic, social, and self-reliance dimensions were also used to measure WE (Jejeebhoy, 1995). In developing the WE index, different empowerment categories include violence against women, employment, education, reproductive healthcare, decision-making, and access to contraceptives (Rettig et al., 2020).

Measuring women's empowerment in both developed and developing countries depends on the data sources (primary or secondary), number of dimensions, and indicators. For Bangladesh, most indexes were created based on the secondary data analysis of DHS data (Sen et al., 2023; Yasmin et al., 2016; Rahman et al., 2021), and only a few studies were conducted using indicators from primary sources (Wei et al., 2021; Winters et al., 2023). Reviews show variations in the use of dimensions and indicators when measuring WE. A summary of the twenty-four studies was presented to show the similarities and dissimilarities of other studies with the proposed research in measuring WE (Table 1). It was seen from the theoretical discussion and review that research considered secondary data, and a limited number of indicators on economic, household, socio-cultural dimensions, and psychological dimensions were almost missing. So, to measure WE, the study focused on the four dimensions of WE with the highest number (33 indicators) of indicators from primary data sources.

**Table 1:** Summary of the research by number dimensions and indicators to measure the WEI.

**SL**	**References**	**Study area/country**	**Data type**	**Dimensions and indicators**	**Total**
	Proposed study of the Authors	Bangladesh	Primary	Economic = 8; Household = 7; Socio-cultural = 8; Psychological = 10	33
1	Sen et al. (2023)	Bangladesh	DHS: 2017–18	Economic = 1; Household = 4; Socio-cultural = 8; Psychological = 0 **Other dimensions** Attitude toward intimate partner violence = 5; Health access barriers = 4	13 + 9
2	Winters et al. (2023)	Bangladesh	Primary	Economic = 5; Household = 7; Socio-cultural = 3; Psychological = 0	15
3	Yasmin et al. (2016)	Bangladesh	DHS: 2011	Economic = 1; Household = 2; Socio-cultural = 1; Psychological = 0	4
4	Rahman et al. (2021)	Bangladesh	DHS: 2017–18	Economic = 0; Household = 5; Socio-cultural = 6; Psychological = 0; Attitudes toward wife-beating = 5	11 + 5
5	Wei et al. (2021)	Bangladesh	Primary	Economic = 5; Household = 5; Socio-cultural = 5; Psychological = 0; Gender Attitude and Beliefs = 5; Relative Freedom from Domination by the Family = 3	15 + 8
6.	Mahmud et al. (2012)	Bangladesh	Primary	Economic = 1; Household = 10; Socio-cultural = 2; Psychological = 16	29
7	Khan et al. (2020)	India	Primary	Economic = 6; Household = 0; Socio-cultural = 7; Psychological = 6; Political = 6	19 + 6
8	Khan et al. (2021)	India	Primary	Economic = 6; Household = 0; Socio-cultural = 7; Psychological = 6; Political = 6	19 + 6
9	Khatiwada et al. (2020)	Nepal	DHS: 2016	Economic = 3; Household = 1; Socio-cultural = 5; Psychological = 0	9
10	Pratley and Sandberg (2018)	Nigeria	DHS: 2013	Economic = 6; Household = 0; Socio-cultural = 12; Psychological = 8	26
11	Dadras et al. (2022)	Afghanistan/Pakistan	PDHS: 2017–18	Economic = 7; Household = 3; Socio-cultural = 7; Psychological = 0; Education = 3; Health = 6	17 + 9
12	Abbas et al. (2021)	Pakistan	DHS: 2012–13 & 2017–18	Economic = 2; Household = 1; Socio-cultural = 1; Psychological = 0; Ownership = 1	4 + 1
13	Ishfaq et al. (2022)	Pakistan	Pakistan Rural Household Panel Survey (PRHPS) (2010–2014)	Economic = 17; Household = 24; Socio-cultural = 11; Psychological = 0; Autonomy = 7; Time Allocation = 3; Qualification = 5; Awareness = 4; Political Empowerment = 6; Violence = 12	52 + 37
14	Hussain and Jullandhry (2020)	Pakistan	Primary	Economic = 8; Household = 9; Socio-cultural = 9; Psychological = 11	37
15	Riddle et al. (2023)	East Africa	DHS: Ethiopia (2016), Kenya (2014), Tanzania (2015–16) and Uganda (2016)	Economic = 3; Household = 4; Socio-cultural = 8; Psychological = 0; Barriers to healthcare = 4; Rejects IPV = 5	15 + 9
16	Miedema et al. (2018)	East Africa	DHS: Ethiopia (2011), Kenya (2014), Rwanda (2010), Tanzania (2010), and Uganda (2011)	Economic = 0; Household = 5; Socio-cultural = 0; Psychological = 0; Human/Social Assets = 9; Gender beliefs and attitudes toward beating justified = 8	5 + 17
17	Elezaj et al. (2019)	Ethiopia	DHS: 2000, 2005, 2011 & 2016	Economic = 1; Household = 4; Socio-cultural = 0; Psychological = 0; Education = 2; Attitudes toward wife-beating = 5	5 + 7
18	UNICEF (2020)	Kenya	DHS 2014	Economic = 2; Household = 4; Socio-cultural = 4; Psychological = 0; Attitudes toward wife beating = 5; Control over sexual relation = 3	10 + 8
19	Mganga et al. (2021)	Tanzania	DHS: 2004–05, 2010, & 2015–16	Economic = 2; Household = 7; Socio-cultural = 6; Psychological = 0; Attitudes toward violence = 5; Age at critical life events = 3; Access to healthcare = 5	15 + 13
20	Atake and Gnakou Ali (2019)	Sub-Saharan Africa	DHS: Burkina Faso (2010), Chad (2014), Mali and Niger (2012)	Economic = 4; Household = 9; Socio-cultural = 3; Psychological = 0	16
21	Asaolu et al. (2018)	Sub-saharan African countries	DHS: 2011, 2014, 2015	Economic = 4; Household = 3; Socio-cultural = 9; Psychological = 0; Education = 3; Health = 6	16 + 9
22	Nyathi and Benhura (2021)	Southern African countries	DHS: Lesotho (2014), Malawi (2015), and Zimbabwe (2015)	Economic = 2; Household = 2; Socio-cultural = 0; Psychological = 0; Fertility = 3; Attitude toward domestic violence = 3	4 + 6
23	Al-Rashdi and Abdelwahed (2022)	Saudi Arabia	Primary	Economic = 4; Household = 0; Socio-cultural = 5; Psychological = 5; Political empowerment = 3; Family support = 5	14 + 8
24	Salem et al. (2020)	Egypt	Primary	Economic = 5; Household = 5; Socio-cultural = 4; Psychological = 0; Gender attitudes = 11	14 + 11

Source: Author's compilation. Blue text represents other dimensions/indicators used in the study.

## Methods

### Data and study area

A cross-sectional study was conducted in eight Mohallas under eight Wards of Dhaka City among randomly selected 625 married women aged 15–49 with at least one child through a face-to-face interview using a semi-structured questionnaire. Probability proportional to size (PPS) sampling was used to determine the number of respondents from each mohalla. Before the start of the main survey, a pre-testing questionnaire was conducted by interviewing a sample of thirty women and addressing the suggestions and problems identified in the questionnaire. Eligible women were selected randomly from the list of eligible women, prepared with the help of the Ward Commissioner office of the respective Mohallas (the smallest geographic area of the City Corporation). The following formula was used to calculate the sample size for the study:


$$\begin{array}{l} n=\;\frac{z^{2}\times pq\times \left(deff\right)}{e^{2\;}\times rr} \end{array}$$

Here, *n* = total sample size; *z* = the standard normal deviation, usually set at 1.96 at a 95% confidence level; *p* = predicted (anticipated) prevalence of women empowerment = 0.50; *q* = 1 – p; deff = Design effect = 1.5; *e*= Margin of error = 0.05; rr = Response rate = 0.90. Using the formula, the sample size was 640. Finally, the study ended with a successful interview with 625 respondents. The non-response rate was 2.34%, and the reasons were busy schedules, reluctance, interaction due to family chores, and concerns about the presence of husbands or in-laws. Informed consent was obtained before the interview commenced. It took 40–45 min to complete the questionnaire.

### Study variables

In total, 33 variables were included in this study under the four dimensions of women's empowerment. Definitions of the economic, familial, sociocultural, and psychological dimensions were as follows:

**Economic:** The capacity of women to acquire and exercise influence over economic resources (Malhotra et al., 2002; Schuler et al., 2010). **Familial:** Women's decision-making capacity to make choices that would significantly positively affect themselves and their families (Malhotra et al., 2002; Duflo, 2012). **Sociocultural:** Freedom for women to use media and technology, as well as to live their lives outside the house (Malhotra et al., 2002; Donta et al., 2016; Sharma and Sanchita, 2016; Singh and Babbar, 2022). **Psychological:** Women's internal sense of empowerment and self-esteem (Malhotra et al., 2002; Sinclair et al., 2010; Rosenberg, 1965). Table 2 presents the details of the dimension-specific indicators, descriptions, and their codes.

**Table 2:** Dimension-specific indicators, descriptions, and codes are used to describe different dimensions of women's empowerment.

**Dimension**	**Independent variable**	
	**Description of the indicators**	**Code**
Economics (**ECO**) (eight indicators)	• Women's control over the family budget (ECO1) • Who usually decides on major HH purchases (ECO2)^*^ • Who usually decides on HH savings (ECO3) • Who usually decides to buy something the respondent needs (ECO4) • Women's control over their own income (ECO6)^*^	• 0 = Can't decide by herself • 1 = Jointly decides with husband • 2 = Decides by herself
	Asset ownership of women (ECO7)^*^	0 = Low (no asset/any one asset) • 1 = Moderate (any two of the assets) • 2 = High (more than two assets)
	Share of household income provided by women (ECO5)	• 0 = No • 1 = < 50% • 2 = More than 50%
	Women involved in income-generating activities (ECO8)^*^	• 0 = No involvement • 1 = Moderate • 2 = High
Household (**HH**) (seven indicators)	Who usually decides on - cooking food for meals (HH1) - inviting guests to your home (HH2) - about the respondent's health care (HH3)^*^ - their own child's healthcare (HH4) - how many children to have (HH5) - when to have a child (HH6) - family planning and contraceptive use (HH7)	• 0 = Can't decide by herself • 1 = Jointly decides withhusband • 2 = Decides by herself
Socio-Cultural (**SCO**) (eight indicators)	Are you allowed to go out to the - local market/bazaar/bank (FM01) - local health center/doctor's clinic (FM02) - home of family/relatives (FM03)^*^ - other cities (FM04)	• 0 = Not at all • 1 = Needs someone toaccompany her • 2 = Yes, can go alone
How frequently do you engage with- - reading newspapers or magazines (ME01) - watching television (ME02) - listening radio (ME03) - using Internet (ME04)	0 = Low access (not at all/rarely) • 1 = Medium access (at leastonce a week; more than once a week/sometimes) • 2 = High (daily)
Psychological (**PHY**) (10 indicators from Rosenberg self-esteem scale (RSES) (Pratley and Sandberg, 2018)	• On the whole, I am satisfied with myself (SE01) • I feel that I have several good qualities (SE03) • I can do things as well as most other people (SE04) • I think that I'm a person of worth, at least on an equal plane with others (SE07) • I take a positive attitude toward myself (SE10)	• 0 = Strongly disagree • 1 = Disagree • 2 = Agree • 3 = Strongly agree
	• At times, I think I am no good at all (SE02RR) • I feel I do not have much to be proud of (SE05RR) • I certainly feel useless at times (SE06RR) • I wish I could have more respect for myself (SE08RR). All in all, I am inclined to feel that I am a failure (SE09RR)	• Reverse coding • 0 = Strongly agree • 1 = Agree • 2 = Disagree • 3 = Strongly disagree

^*^Variables used in BDHS 2017–18. In addition to these six variables, a few researchers used 5–10 additional variables from BDHS as proxy indicators for WEI.

### Statistical analysis

The questionnaire's reliability was assessed by using Cronbach's alpha. Both exploratory (EFA) and confirmatory factor analysis (CFA) were used to identify the possible underlying factors and verify the factor structure (Cabrera-Nguyen, 2010; Norris and Lecavalier, 2010; Hair et al., 2019; Field, 2013). To test the construct validity, using STATA's “split sample command,” we randomly split the data into two parts: one for EFA and the other for CFA (Cabrera-Nguyen, 2010). The Kaiser Meyer Olkin (KMO) test of sampling adequacy and the Bartlett test of sphericity validated the data for EFA (Kaiser, 1974; Stevens, 2002). The number of retained factors was determined using three criteria (Hair et al., 2019) -the cumulative percentage of variance extracted, Kaiser's criteria (eigenvalue > 1 rule) (Kaiser, 1974), and the scree test (Cattell, 1966). Items loaded on more than one factor or < 0.5 were removed (Hair et al., 2019; Field, 2013; Cattell, 1966). After EFA, we conducted CFA on the other half of the sample to validate the EFA-derived factors (Cabrera-Nguyen, 2010; Dadras, 2015). Confirmatory factor analysis (CFA) includes considerations for measurement and structural models. To assess the goodness of fit of the model, absolute, incremental, and parsimony fit indices such as chi-square (2) statistics, chi-square/df (CMIN/DF), the root mean squared error of approximation (RMSEA), Comparative Fit Index (CFI), Tucker-Lewis Index (TLI), and Normed Fit Index (NFI) are used (Field, 2013). The factor loading for each item on its latent variable, which should be >0.5, and composite reliability (CR), which should be >0.7, was used to estimate the measurement model's validity and reliability (Fornell and Larcker, 1981). Convergent and discriminant validity were also assessed during CFA. Convergent validity can be determined via factor loadings, average variance extracted (AVE), and composite reliability (Fornell and Larcker, 1981). Factors loading more than 0.5 reflect the evidence of convergent validity. On the other hand, AVE should be more than 0.50, meaning all the latent variables account for more than 50% of the overall variance. The composite reliability for all the constructions is ~ 0.7 or higher than 0.7.

The Fornell-Larcker criterion and Heterotrait-Monotrait Ratio (HTMT) were used to assess the discriminant validity (Fornell and Larcker, 1981). To ensure distinctiveness between variables, the HTMT must not exceed 0.9 (Henseler et al., 2015). Data were analyzed using SPSS v 22 and AMOS version 24.

## Results

### Socio-demographic characteristics of the respondents

Among the respondents, 36.0% were aged 15–29, and 32.0% had completed their secondary education. A notable number of respondents, 226, belonged to people with middle incomes (36%) in wealth, and the highest number of women (64.0%) were unemployed. In addition, 39% of the respondents married before turning 18. On average, only one member earns money, and the average family income is ~BDT 38,000 (Table 3).

**Table 3:** Sociodemographic characteristics of 625 married women in a sample in Dhaka, Bangladesh.

**Characteristics**	**Frequency**	**Percentage**
**Age of the respondent**		
< 30	226	36
30–39	219	35
40+	180	29
**Respondent's education**		
Illiterate	98	16
Primary	136	22
Secondary	200	32
Higher Secondary or more	191	31
**Wealth index**		
Poor	204	33
Middle	226	36
Rich	195	31
**Religion**		
Non-Muslim (R)	115	18
Muslim	510	82
**Age at marriage**		
Before 18	244	39
At 18 or above	381	61
**Respondent's occupation**		
Not working	398	64
Working	227	36
Average family income (BDT) (1$ = BDT. 115.0 in April 2024	38,000	
Average earning member	1.42	

### Multivariate analysis

**Figure 1:**
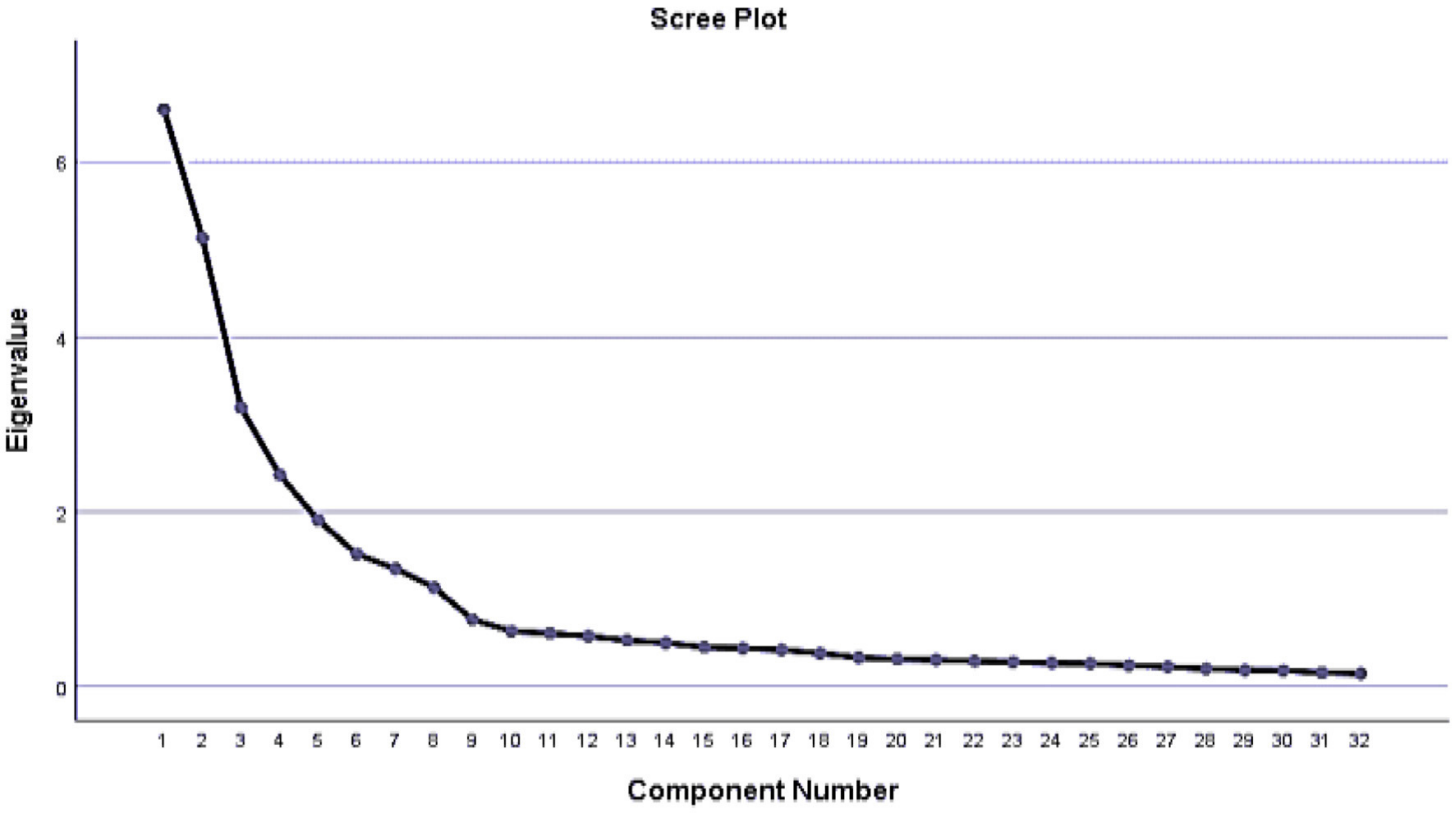
Scree plot for dimensions of overall empowerment.

In addition to the correlation matrix showing an appropriate correlation, the study's KMO value was 0.832, indicating adequate data. Furthermore, the Bartlett test of sphericity was significant at a *p*-value < 0.001, which supports the suitability of data for EFA. Initially, thirty-three items were included in the EFA. Since all the factors had more than three items except ME03: “frequency of listening radio,” the ME03 item was eliminated from the analysis. The final model consisted of thirty-two items, which loaded on eight factors with eigenvalues (>1) and explained 72.82% of the variation in the data (Table 4). The calculated communality value is closer to 1, indicating that the factors explain the variable well. Figure 1 depicts a scree plot that shows the eight extracted constructs for women's empowerment.

**Table 4:** Dominant dimensions of women empowerment.

**Dimensions**	**Code**	**Component (Factors)**								**Variation (%)**	**Communalities**
		**1**	**2**	**3**	**4**	**5**	**6**	**7**	**8**		
PHY	SE07	0.884								20.2	0.827
	SE04	0.871									0.798
	SE03	0.866									0.837
	SE10	0.859									0.775
	SE01	0.775									0.750
PHY	SE06RR		0.839							16.0	0.764
	SE05RR		0.834								0.739
	SE08RR		0.833								0.721
	SE09RR		0.701								0.634
	SE02RR		0.679								0.551
ECO	ECO6			0.856						10.2	0.793
	ECO8			0.856							0.783
	ECO5			0.846							0.796
	ECO7			0.618							0.586
	SCO	FM02				0.857					7.5	0.820
FM03					0.834						0.806
FM04					0.770						0.625
FM01					0.754						0.620
ECO		ECO3					0.852				6.3	0.759
	ECO4					0.808					0.739
	ECO2					0.739					0.756
	ECO1					0.709					0.660
	HH	HH1						0.842			4.7	0.742
HH2							0.771				0.693
HH4							0.743				0.641
HH3							0.612				0.617
HH		HH6							0.872		4.4	0.826
	HH5							0.834			0.798
	HH7							0.719			0.588
	SCO	ME04								0.847	3.3	0.807
		ME02								0.813		0.755
ME01									0.727		0.647

ECO, economic; HH, household; ME, media exposure; FM, freedom of movement; SE, self-esteem.

Table 4 shows that the analysis categorized similar factors together and divided the four dimensions of empowerment into eight factors. The first 20.2% and second 16.0% factors indicated positive self-esteem (SEP) and negative self-esteem (SEN) (reverse coded), accounting for the most significant portion of variations of the final model. The items loaded on the third (10.2%) and fifth factors (6.3%) indicated economic empowerment and control over economic decision-making. The fourth and eighth factors indicated socio-cultural empowerment (labeled as women's movement and women's access to information and technology). The statements loaded on the sixth and the seventh factors indicated household empowerment (labeled as household decision-making and participation in reproductive discussion).

Confirmatory factor analysis (CFA) is subsequently conducted to test and validate the identified factor structure from EFA (Table 4). CFA assessed the model with the help of various goodness of fit indices. Results of the CFA model support good model fit: Chi-square (χ^2^) statistics is 865.513, chi-square/df (CMIN/DF) is 1.994, RMSEA is 0.057, CFI is 0.926, TLI is 0.916, and NFI is 0.921. All the items have sufficient loadings on their latent construct (>0.5 or, ideally, >0.7). This supports the idea that convergent validity is achieved in all dimensions. The construct reliability value for all four constructs exceeds the threshold level of 0.7. All square correlation values were lower than the AVE values of their respective factors, indicating no issue in the model's discriminant validity.

The correlation matrix (Table 5) shows that SEP and SEN have a higher association among all other factors. Similarly, COED and EI have a greater correlation (0.647); FM and ME have a moderate correlation (0.538); and HDM and RDM have a higher correlation (0.650). The high correlation between the two latent variables provides the basis that these two sub-dimensions can be used to construct individual dimensions such as economic, household, sociocultural, and psychological dimensions. The high correlation between the two latent variables indicates that they have griped similar features or manifestations of the underlying construct. The measurement model is simplified by reducing the number of latent variables and making the model more concise and easier to understand.

**Table 5:** Correlation among latent constructs.

			**Estimate**				**Estimate**
SEP	< ->	SEN	**0.519**	COED	< ->	ME	0.189
SEP	< ->	COED	0.072	COED	< ->	RDM	0.102
SEP	< ->	FM	0.125	FM	< ->	HDM	0.196
SEP	< ->	HDM	0.249	FM	< ->	EI	0.255
SEP	< ->	EI	−0.058	FM	< ->	ME	**0.538**
SEP	< ->	ME	0.273	FM	< ->	RDM	0.261
SEP	< ->	RDM	0.122	HDM	< ->	EI	0.103
SEN	< ->	COED	0.000	HDM	< ->	ME	0.239
SEN	< ->	FM	0.085	HDM	< ->	RDM	**0.650**
SEN	< ->	HDM	0.174	EI	< ->	ME	0.165
SEN	< ->	EI	−0.103	EI	< ->	RDM	0.246
SEN	< ->	ME	0.221	ME	< ->	RDM	0.226
SEN	< ->	RDM	0.050				
COED	< ->	FM	0.284				
COED	< ->	HDM	0.073				
COED	< ->	EI	**0.647**				

EI, economic independence; COED, control over economic decision making; HDM, household decision making; RDM, reproductive decision making; FM, freedom of movement; ME, media exposure; SEP, self-esteem positive SEN; SEN, self-esteem negative. Bold values indicate a higher correlation.

**Figure 2:**
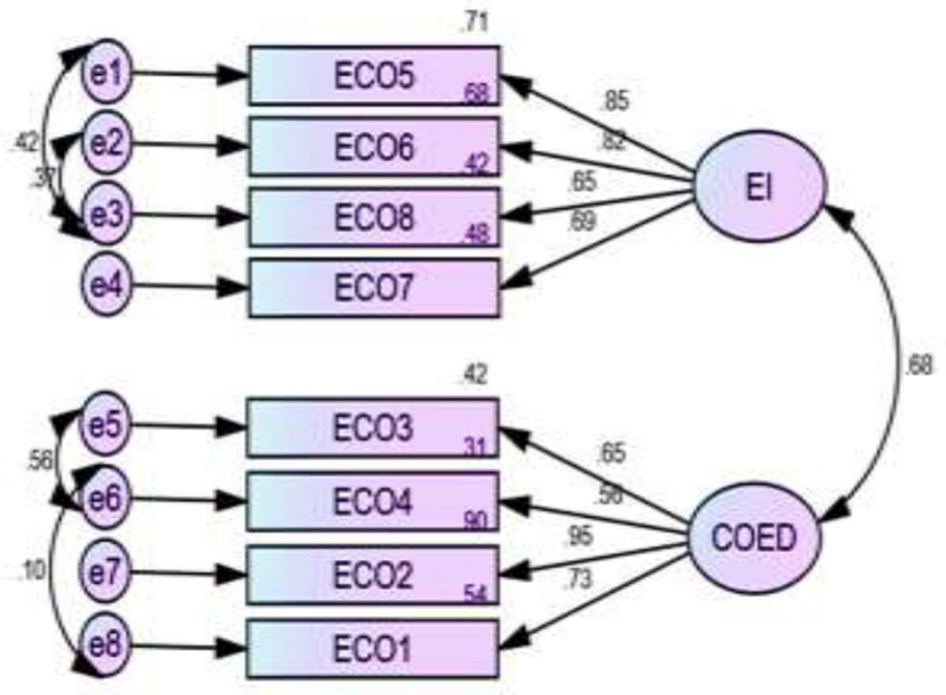
Two factors measure economic empowerment and their relevant items, where EI, economic independence; COED, control over economic decision making.

**Figure 3:**
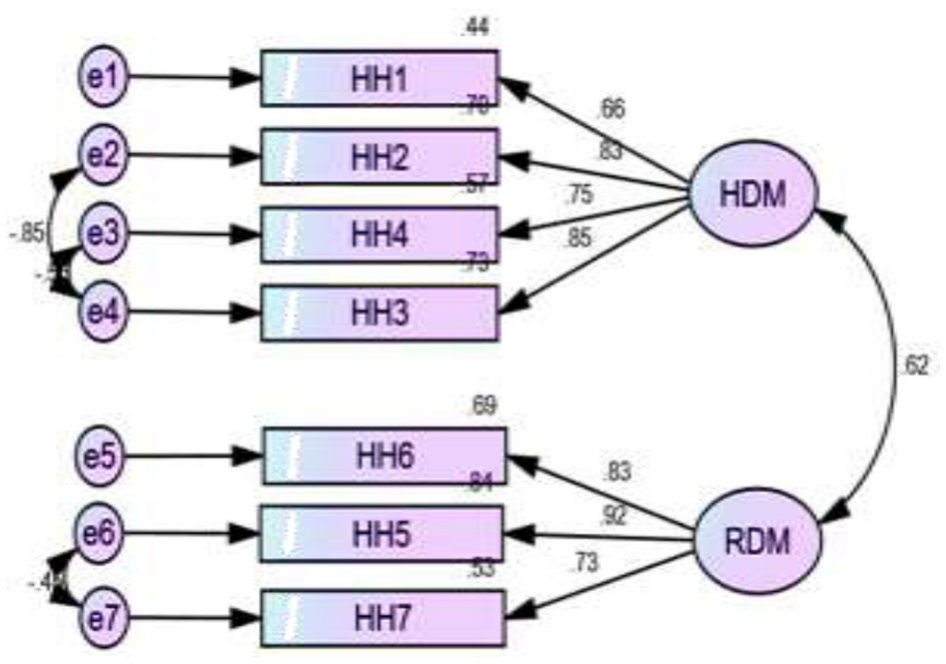
Two factors measure women's empowerment within the household and their relevant items, where HDM, household decision making; RDM, reproductive decision making.

**Figure 4:**
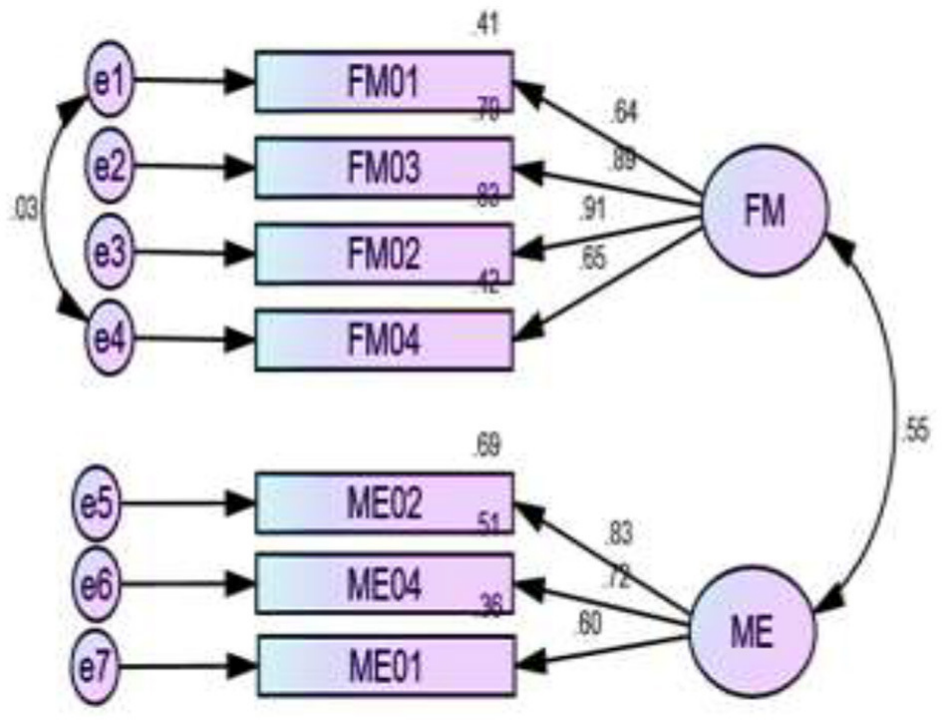
Two factors measure sociocultural empowerment and their relevant items. FM, freedom of movement; ME, media exposure.

**Figure 5:**
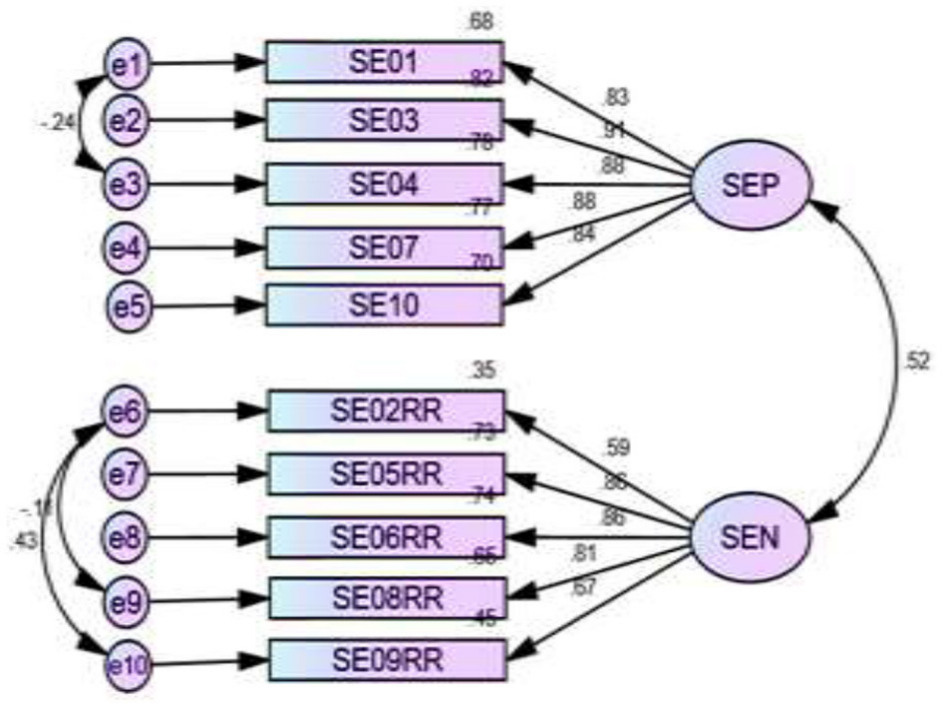
Two factors measure psychological empowerment and their relevant items. SEP, self-esteem positive SEN; SEN, self-esteem negative.

Figures 2–5 depict the path diagram for each dimension. Four CFA path diagrams show that each item has sufficient loadings on its latent construct (>0.5 or, ideally, >0.7). The goodness of fit indices (Table 6) and the composite reliability (CR) value (Table 7) confirm convergent and discriminatory validity in all the dimensions, which ensures the construct validity of the models.

**Table 6:** Results for fit indices.

**Fit index**	**Results of the present study**			
	**ECO**	**HH**	**SOC**	**PHY**
Comparative fit index (CFI)	0.986	0.983	0.983	0.975
Goodness of fit index (GFI)	0.978	0.980	0.980	0.958
Adjusted Goodness of Fit Index (AGFI)	0.947	0.945	0.953	0.925
Normed fit index (NFI)	0.981	0.978	0.978	0.968
Incremental Fit Index (IFI)	0.986	0.983	0.983	0.975
Root Mean Square Error of Approximation (RMSEA)	0.064	0.073	0.067	0.076
Chi-square normalized by degrees of freedom	3.593	4.349	3.826	4.558

ECO, economic empowerment; HH, household empowerment; SOC, sociocultural empowerment; PHY, psychological empowerment.

**Table 7:** Reliability and validity analysis.

**Dimension**	**Code**	**Indicator**	**Square root of AVE**	**CR**	**AVE**
Economic (ECO)	COED	ECO1	0.735	0.819	0.541
		ECO2			
		ECO3			
		ECO4			
	EI	ECO5	0.747	0.833	0.558
		ECO6			
		ECO7			
		ECO8			
Familial (HH)	HDM	HH1	0.761	0.846	0.580
		HH2			
		HH3			
		HH4			
	RDM	HH5	0.824	0.862	0.680
		HH6			
		HH7			
Socio-cultural (SOC)	FM	FM01	0.787	0.864	0.619
		FM02			
		FM03			
		FM04			
	ME	ME01	0.751	0.793	0.564
		ME02			
		ME04			
Psychological (PHY)	SEP	SE01	0.859	0.933	0.737
		SE03			
		SE04			
		SE07			
		SE10			
	SEN	SE02RR	0.752	0.864	0.565
		SE05RR			
		SE06RR			
		SE08RR			
		SE09RR			

EI, economic independence; COED, control over economic decision making; FM, freedom of movement; ME, media exposure; HDM, household decision making; RDM, reproductive decision making; SEP, self-esteem positive; SEN, self-esteem negative.

Table 6 presents the various parameters used to check the goodness of fit in CFA. All the items under each dimension are well-fitted.

Table 7 shows the model validation results (measurement model). The Composite reliability (CR) (>0.7) and Average Variance Extracted (AVE) (>0.5) values provide evidence that all the individual CFA models are reliable.

## Discussion

In this study, our main objective was to revisit and validate the women's empowerment index for Bangladesh by adding additional indicators and psychological dimensions. This study utilized exploratory and confirmatory factor analysis to establish a reliable and valid four-dimensional construct for measuring women's empowerment in Bangladesh. This reduces vagueness in quantitatively conceptualizing and operationalizing empowerment. The four dimensions are economic, household, socio-cultural, and psychological.

Prior studies across Sub-Saharan Africa have conceptualized women's empowerment using four domains (Huis et al., 2017; Miedema et al., 2018; Asaolu et al., 2018). In Bangladesh, three research (Sen et al., 2023; Yasmin et al., 2016; Rahman et al., 2021) used four indicators, and Rahman et al. (2021) used 16 indicators based on BDHS-2011 and BDHS-2017/18. Few studies (Wei et al., 2021; Winters et al., 2023) have used primary data to construct a women empowerment index in Bangladesh. However, these studies used different methods. The current study found a valid and reliable Bangladeshi-specific index for women's empowerment composed of four dimensions and eight domains measured by 32 items.

Under four dimensions there were eight domains from this study namely, economic independence, control over household financial decisions, household decision-making, reproductive decision-making, freedom of movement, media exposure, positive self-esteem, and negative self-esteem that were similar to those identified by Richardson (2018), Asaolu et al. (2018), Miedema et al. (2018), Zeisler (2017), Karimli et al. (2021), Duflo (2012), Asiedu et al. (2021), Febro et al. (2021), Gnambs and Schroeders (2020), and Ewerling et al. (2017).

In Bangladesh, our findings underscore the importance of positive self-esteem in women's empowerment, followed by negative self-esteem as the second most important domain. These domains are integral components of psychological empowerment. This aligns with existing literature emphasizing the significance of psychological empowerment as a key factor in promoting women's empowerment (Huis et al., 2017; Khan et al., 2020; Moubarak et al., 2022). A study by Mahmud et al. (2012) revealed that out of two self-esteem indicators (beating is not justified and the number of household decisions), women are most likely to feel empowered concerning household decision-making, and one self-esteem indicator. This underscores the significance of fostering positive self-esteem and empowering women in household decision-making.

“Economic independence” as a composite measure based on several indicators, including asset ownership, the share of household income provided by women, women's involvement in income-generating activities, and their control over their income, emerged as the domain of economic empowerment dimensions. This conceptualization aligns with previous research (Malhotra et al., 2002; Phan, 2016; Wei et al., 2021; Asaolu et al., 2018; Zeisler, 2017; Karimli et al., 2021; Deutsch and Silber, 2019) that has recognized these components as key factors contributing to women's economic empowerment and autonomy. Kabeer (1999) defines empowerment as a woman's “ability to define goals and act upon them”. Women's participation in household decision-making, alone or jointly, can lead to greater investment in children's education and health, including reproductive health (Winters et al., 2023). Reproductive decision-making is also an important factor in this study.

Freedom of movement was another key domain defining women's empowerment in Bangladesh. According to Mahmud et al. (2012), women's mobility indicator determines the extent to which women can go outside the home and their autonomy in terms of not being required to seek consent from their husbands or any other household member. Women's freedom of movement and access to information and technology, both domains used to measure socio-cultural empowerment in the current study, is supported by several studies (Wei et al., 2021; Asiedu et al., 2021; Febro et al., 2021).

The present study's findings support the multidimensional construct of women's empowerment. Literature also supports using economic, familial, sociocultural, and psychological dimensions to measure women's empowerment collectively (Jejeebhoy and Sathar, 2001). These dimensions encompass various factors that contribute to women's overall empowerment. This study strongly focuses on developing a comprehensive and reliable method for measuring women's empowerment in quantitative research conducted in Bangladesh. The validity of the data was displayed through the findings of confirmatory factor analysis, which showed optimal performance of the index across four distinct dimensions using the measures of parsimony (RMSEA) and ft (CFI & SRMR). Furthermore, utilizing a primary data set has enabled this study to elucidate the significance of women's empowerment in Bangladesh. Though BDHS addressed the issue of women's empowerment, they considered a limited number of indicators. An important strength of this study lies in the validated measurement model and the identification of key dimensions, which provide a solid foundation for designing effective policies and programs. Also, the empowerment index assists as a significant tool for predicting various behaviors, particularly in areas such as health, education, and poverty reduction. It emphasizes the importance of empowerment in promoting positive changes in society by providing insights into how various dimensions of empowerment influence the decisions and actions of individuals. By comprehending the different levels of empowerment, policymakers and practitioners can formulate specific interventions that promote desired behaviors, resulting in enhanced outcomes in sectors such as healthcare utilization, educational achievement, and overall wellbeing (Rahman et al., 2021; Wei et al., 2021; Winters et al., 2023).

However, this study does have some limitations. The components that are used to construct the women's empowerment index rely on self-reported data from women, which may be susceptible to social desirability bias. We did not gather parallel responses from husbands. However, we recognize the need to employ triangulation to verify the accuracy and reliability of the wives' responses. The current study has limitations, and there is potential for future research to include a comparative analysis of husbands' reactions. Furthermore, the factors used to evaluate the empowerment of women fluctuate with time and context. These factors may be significant in each context when they are in their initial stages, but they become less significant or useful as they become normative. Furthermore, psychological empowerment has several features, including self-esteem, self-confidence, psychological wellbeing, self-determination, self-awareness, positive thinking, and happiness. In this study, we considered only self-esteem as a measure of psychological empowerment.

## Conclusion

This study revisits and updates the measurement tools used to assess women's empowerment and ensures that the indicators used to measure this complex idea are valid and reliable. The research is specifically tailored to the context of Bangladesh, creating a dimension-specific measure of women's empowerment. By validating the factor structure through CFA, we offer a reliable measurement model that contributes to the existing literature on women's empowerment. In this study, we got eight factors under a four-domain construct, each composed of two factors. In contrast to previous research that mostly focused on economic, household, and sociocultural factors, this study includes psychological dimensions to the empowerment index by examining both positive and negative aspects of self-esteem. This addition reflects a deeper understanding of the internal attributes of empowerment that affect women's autonomy and agency.

The factors we evaluated to promote women's empowerment have been proven. The study confirms the reliability and accuracy of the measures by validating the items used to measure the various dimensions of women's empowerment. This finding will aid in reducing uncertainty in conceiving and operationalizing empowerment based on empirical research conducted in Bangladesh. Dimension-specific measures can provide valuable insights into the status of women's empowerment in four domains: economic, household, sociocultural, and psychological. As a result, academics and policymakers can make rational decisions regarding the elements that lead to women's empowerment in Bangladesh and devise effective interventions and policies to promote gender equality. These measures can help identify areas where women's empowerment needs strengthening and guide the development of effective policies and programs. By revisiting the dimensions of women's empowerment, the study aims to contribute to the existing knowledge by providing updated insights, identifying any new dimensions, and assessing the effectiveness of past strategies in promoting women's empowerment in Bangladesh.

## Data Availability

The raw data supporting the conclusions of this article will be made available by the authors, without undue reservation.
